# From targets to solutions: Implementing a trauma quality improvement bundle in Cameroon

**DOI:** 10.1016/j.injury.2024.111625

**Published:** 2024-05-19

**Authors:** Dennis J. Zheng, Mark T. Yost, Lidwine N. Mbuh, Mirene Tchekep, Jean Baptiste Boumsong, Jean Gustave Tsiagadigui, Rasheedat Oke, Catherine Juillard, Alain Chichom-Mefire, S. Ariane Christie

**Affiliations:** a Program for the Advancement of Surgical Equity, Department of Surgery, University of California Los Angeles, 10833 Le Conte Ave, Suite 7V-111 CHS, Los Angeles, CA 90095, United States of America; b University of Buea, P.O. Box 63, Buea, Cameroon; c Edea Regional Referral Hospital, P.O. Box 100, Edea, Cameroon

**Keywords:** Global surgery, Injury, Quality improvement, Sub-Saharan Africa, Emergency medicine, Trauma surgery

## Abstract

**Background:**

Global surgery research efforts have been criticized for failure to transition from problem identification to intervention implementation. We developed a context-appropriate trauma quality improvement (TQI) bundle to ameliorate care gaps at a regional referral hospital in Cameroon. We determined associations between bundle implementation and improvement in trauma resuscitation practices.

**Methods:**

We implemented a TQI bundle consisting of a hospital-specific trauma protocol, staff training, a trauma checklist, provision of essential emergency trauma supplies in the resuscitation area, and monthly quality improvement meetings. We compared trends in target process measures (e.g., frequency and timing of vital sign collection and primary survey interventions) in the six-month period pre- and post-bundle implementation using Wilcoxon rank-sum and Fisher’s exact tests.

**Results:**

We compared 246 pre-bundle patients with 203 post-bundle patients. Post-bundle patients experienced a greater proportion of all vital signs collected compared to the pre-intervention cohort (0 % pre-bundle vs. 69 % post-bundle, *p* < 0.001); specifically, the proportion of respiratory rate (0.8 % pre-bundle vs. 76 % post-bundle, *p* < 0.001) and temperature (7 % pre-bundle vs. 91 % post-bundle, *p* < 0.001) vital sign collection significantly increased. The post-bundle cohort had vital signs measured sooner (74 % vital signs measured within 15 min of arrival pre-bundle vs. 90 % post-bundle, *p* < 0.001) and more frequently per patient (7 % repeated vitals pre-bundle vs 52 % post-bundle, *p* < 0.001). Key primary survey interventions such as respiratory interventions (1 % pre-bundle vs. 8 % post-bundle, *p* < 0.001) and cervical collar placement (0 % pre-bundle vs. 7 % post-bundle, *p* < 0.001) also increased in the post-bundle cohort.

**Conclusions:**

The implementation of a context-appropriate TQI bundle was associated with significant improvements in previously identified trauma care deficits at a single regional hospital. Data-derived interventions targeting frontline capacity at the local level can bridge the gap between identifying care limitations and improvement in resource-limited settings.

## Background

Global surgery has grown immensely as an academic discipline with the shifting landscape of global health interventions from communicable to non-communicable diseases. However, global trauma quality improvement (TQI) practices remain limited by the gaps that exist between the description of problems and the implementation of solutions [1]. Injury comprises a significant burden of morbidity and mortality in low- and middle-income countries (LMICs) [2,3]. Improvements in trauma care systems in LMICs may save an estimated two million lives each year [4]. Barriers such as prohibitive cost and incompatibility with local capacities have limited the feasibility of implementing tools like advanced trauma life support (ATLS) to improve the quality of trauma care rendered in LMIC settings [5–7].

Cameroon is a majority Francophone lower-middle income country in Central Africa with a high disease burden of injury and a median life expectancy of 58 years of age, which is two years lower than surrounding Central African countries [8–12]. A recent death review conducted within a larger Cameroonian TQI initiative determined nearly one-third of deaths to be preventable or potentially preventable [13]. Evaluation of Cameroon Trauma Registry (CTR) data found that many deaths were associated with underperformance of timely and complete primary survey assessments, as 11 % of in-hospital deaths had no vital signs collected [13]. Likewise, indicated interventions to treat abnormalities found on primary survey, such as intubation, chest tube placement, or cervical collar placement were performed less than 10 % of the time [13]. Based on these findings, an intervention to improve the quality of evaluation and treatment during the primary survey had the potential to improve overall trauma care delivery and decrease preventable deaths. This study examined data regarding local clinical practice patterns in Cameroon and worked with trauma care providers at a regional referral hospital in Cameroon to design a TQI bundle. After implementation of the TQI bundle at this hospital, we evaluated changes in hospital primary survey assessment and intervention process measures as recorded by the CTR.

This TQI bundle intervention served as a first step towards developing a scalable, context-appropriate trauma care bundle throughout Cameroon. We hypothesized that introduction of the TQI bundle would increase the frequency of vital sign collection and improve treatment clinical resuscitation priorities identified on primary survey. We evaluated associations between bundle implementation and changes in trauma resuscitation practices.

## Methods

### Study design

We conducted a pre- and post-intervention observational cohort comparative study using prospectively collected CTR data from injured patients who presented to a single regional hospital in the Littoral region of Cameroon between October 2021 and November 2022.

### Setting

We implemented the study intervention at a public regional referral hospital in the Littoral region of Cameroon. The regional hospital in this study collects trauma patient data for the CTR and serves a catchment area of approximately 100,000 to 300,000 people [14]. The hospital is located along Cameroon’s busiest highway that is a known high-incidence road traffic injury (RTI) corridor. This regional referral hospital contains about 100 beds and has an emergency department in addition to an intensive care unit (ICU) and operating rooms. Medical physicians, general surgeons, and orthopedic surgeons are available to assess and treat injured patients. Anesthesia providers are available for intubation and ventilation of injured patients, but there are no mechanical ventilators available. While blood transfusion is a theoretically available treatment, Cameroon often lacks sufficient blood products due to low blood donor participation rates [15–18]. Available on-site imaging capabilities for injured patients includes X-ray, ultrasound, and computed tomography (CT) scan.

### Participants and data sources

The CTR is a prospective, multisite trauma registry that collects data on patients who present with injury at affiliated facilities throughout Cameroon [10,11,14]. The CTR collects injured patient data from presentation until hospital discharge. We extracted CTR data for patients of all ages who presented to the single regional hospital during the study period to evaluate data on clinical care patterns.

### Study intervention

The TQI bundle components consisted of 1) a hospital-specific trauma protocol 2) a bedside trauma checklist (Supplementary Materials [SM] 1), 3) staff training on protocol implementation, 4) reorganization and provision of essential emergency trauma supplies for rapid access in the resuscitation area (Full list of supplies in SM 2), and 5) monthly meetings with trauma staff to review performance. We based the bedside trauma checklist on the World Health Organization (WHO) Trauma Care Checklist [19]. Additional principles derived from other trainings including the Primary Trauma Care, Kampala Advanced Trauma Care, and WHO Basic Emergency Care courses were incorporated into the checklist [20–22].

The TQI bundle was developed within a larger TQI study as a sitespecific intervention to improve overall quality of trauma care. Data patterns specific to the regional hospital in this study were used to guide selection of TQI bundle components. We used the capability, opportunity, and motivation behavior system (COM-B) and its associated behavior change wheel (BCW) framework in the design of the overall TQI bundle and its components [23]. During this process, we outlined the specific factors influencing provider behaviors during trauma care and aimed possible interventions at each factor (SM 3). Prospective bundle components were then presented to hospital trauma care stakeholders such as surgeons, emergency department (ED) physicians, and nurses. We then collected feedback regarding the utility of each bundle component in group discussions. Iterative cycles of bundle optimization based on stakeholder feedback were performed until we reached majority consensus on each included bundle component. For instance, we designed the bedside trauma checklist specifically to remind providers about the aspects of care identified as deficient through data analysis and regular audit meetings. Prior to implementation of the TQI bundle, patients who presented to the ED could only receive care after the patient or the patient’s family purchased necessary supplies such as intravenous fluid and/or bandages at the hospital pharmacy and brought them to the ED. The hospital staff identified this practice as a major barrier to receipt of timely patient care. Thus, as part of the TQI bundle, the intervention organized sealed bags of essential supplies needed for the emergency care of an individual patient and securely stored them in the ED. Though these supplies helped streamline the process of procuring and utilizing supplies for injury care, the cost of these supplies (approximately 5200 Central African Francs, about $8.70 US dollars) was added to the patient’s hospital bill. of equipment for providers. We used the Standards for Quality Improvement Reporting Excellence (SQUIRE 2.0) guidelines in reporting the background, methods, results, and discussion of this quality improvement intervention [24].

### Statistical methods and data analysis

We compared patterns of clinical trauma care for CTR patients in the six months before bundle implementation against CTR patients enrolled in the six months following bundle implementation. The pre-bundle period lasted from October 2021 until March 2022. During April and May 2022, the TQI bundle iterative review process finalized the components of the bundle. The TQI bundle was implemented in June 2022 and post-bundle data was collected until end of November 2022. Primary variables of evaluation included the completion of primary survey assessment and performance of indicated medical interventions. We examined additional CTR data such as patient demographics, injury characteristics, and disposition outcomes including trauma death.

Categorical variables were reported as frequencies and percentages while continuous variables were reported as median and interquartile range (IQR), respectively. Differences between pre- and post-bundle cohorts were evaluated with the Wilcoxon rank-sum test for continuous variables and 2 × 2 Fisher’s exact test for categorical variables, respectively. We calculated probability of the two-tailed Fisher’s exact test by doubling the exact one-tailed probability, as previously described [25]. For all analyses, an alpha level of less than 0.05 was considered statistically significant. Statistical analysis was performed using Stata version 16 [26].

### Ethics

Approval to conduct this study was granted by institutional review boards (IRBs) (IRB #: #19-000086 and 2021/1506-07/UB/SG/IRB/FHS) and the Cameroon National Ethics Committee (N°2018/09/1094/CE/CNERSH/SP). Patients enrolled in the study were approached by trained Cameroonian research assistants for informed verbal consent. The need for written informed consent for adults and parents/guardians was waived by the IRB. Parents and/or guardians provided verbal consent for patients under the age of 18 years old. Research assistants documented obtaining verbal consent on the registry form.

## Results

The study collected data on 246 injured patients in the six months prior to TQI bundle implementation and 203 patients after implementation (Table 1). Injured patients were usually young (median age 31 years pre-bundle vs. 34 years post-bundle, *p* = 0.006) and predominantly male (75 % male pre-bundle vs. 78 % male post-bundle, *p* = 0.590). There were no significant differences in road traffic injury (RTI) mechanism frequency and injury severity as measured by the highest estimated abbreviated injury scale (HEAIS) [27] in the pre- and post-bundle cohorts. A similar proportion of patients were admitted to the ward or ICU after bundle implementation (30 % pre-bundle vs. 32 % post-bundle, *p* = 0.736). However, a smaller percentage of patients in the post-bundle cohort were admitted directly to the operating room (7 % pre-bundle vs. 4 % post-bundle, *p* = 0.030). ED mortality was 3 % (*n* = 8) before and 3 % (*n* = 5) after bundle implementation (*p* = 0.818).

**Fig. 1.**
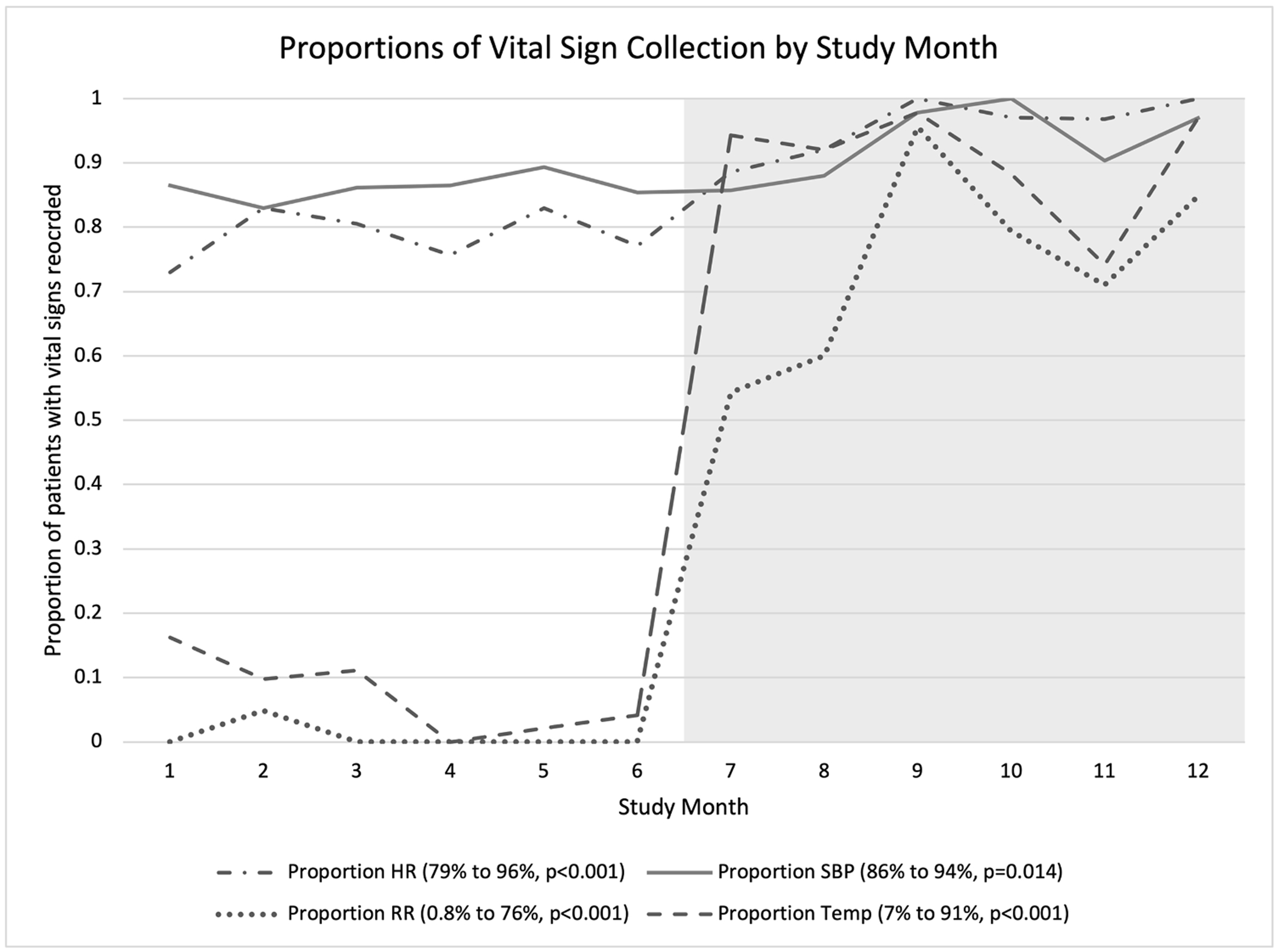
Proportions of vital sign collection pre- and post-bundle intervention. The p-value in Fig. 1 refers to statistical significance in the difference of percentages of patients who experienced individual vital sign (e.g., heart rate, blood pressure, respiratory rate, temperature) measurement in the 6 months before implementation compared to the 6 months after implementation of the TQI-bundle. The differences in the percentages were compared via chi-square analysis. The gray area of the figure represents the post-bundle implementation period. HR = heart rate, SBP = systolic blood pressure, RR = respiratory rate, Temp = temperature.

Vital sign collection improved significantly in the post-bundle cohort. While zero patients in the pre-bundle cohort had a complete set of initial vitals (e.g., heart rate, blood pressure, respiratory rate, and temperature) collected, 69 % (*n* = 140) of post-bundle patients had a complete set of vitals recorded (*p* < 0.001) (Table 1). All vital sign collection improved in the post-bundle cohort (Fig. 1). A greater proportion of patients (96 %, *n* = 195) in the post-bundle cohort had heart rate collected compared to 79 % (*n* = 194) of the pre-bundle patients (*p* < 0.001). Similarly, a larger proportion of patients experienced blood pressure measurements after the bundle implementation (86 %, *n* = 212 pre-bundle vs. 94 %, *n* = 190 post-bundle, *p* = 0.014). The collection of both respiratory rate (0.8 %, *n* = 2 pre-bundle vs. 76 %, *n* = 154 post-bundle, *p* < 0.001) and temperature (7 %, *n* = 17 pre-bundle vs. 91 %, *n* = 185 post-bundle, *p* < 0.001) vital signs increased with the TQI bundle implementation. Additionally, median time to initial vital sign measurement significantly decreased in the post-bundle cohort (5, IQR 2–8 min pre-bundle vs. 3, IQR 0–5 min post-bundle, *p* < 0.001). The post-bundle cohort also experienced less delays in vital sign collection, as over 90 % (*n* = 183) of patients had vital signs recorded within 15 min of presentation compared to 74 % (*n* = 181) of pre-bundle patients (*p* < 0.001). Furthermore, serial measurement of heart rate and blood pressure occurred in 52 % (*n* = 106) of post-bundle patients compared to only 7 % (*n* = 17) of patients in the pre-bundle cohort (*p* < 0.001).

Moreover, the post-bundle cohort experienced significant improvements in the recognition and treatment of key primary survey abnormalities such as airway (e.g., non-patent airway) problems (Table 2). Patients in the post-bundle cohort experienced a greater than five-fold increase in breathing interventions during the primary survey (1 %, *n* = 3 pre-bundle vs. 8 %, *n* = 16 post-bundle, *p* < 0.001). Cervical spine collars were placed more frequently in the emergency department among post-bundle patients (0 % pre-bundle vs. 7 %, *n* = 14 post-bundle, *p* < 0.001). The number of used pre-packaged trauma kits ranged from 0 to 14 kits used per week (SM 4). The large variation in the number of utilized was influenced by the inconsistent availability of supplies in the hospital pharmacy each week.

## Discussion

In this study, we evaluated the changes in practice patterns before and after implementation of a context-appropriate, data driven TQI bundle at a regional referral hospital in Cameroon. Implementing the TQI bundle was associated with significant improvements in trauma practice measures such as completeness and timeliness of vital sign collection and frequency of primary survey breathing interventions. Providers demonstrated sustained improvement in fundamental primary survey practices over six months after TQI bundle implementation. We expect these continued practices to result in earlier identification and treatment of critical injuries, ultimately improving overall trauma outcomes at the institution.

Trauma care interventions in LMICs that seek to mitigate poor outcomes due to care gaps often are limited by cost constraints, resource scarcity, and lack of protected time for qualified instructors [5–7,28,29]. The TQI bundle in this study collaborated with local partners and stakeholder to focus on data review, training, and improving timely access to existing resources. This study demonstrated that a low-cost intervention that utilizes existing resources can result in measurable improvements in fundamental trauma care delivery. However, the intervention must be evidence-based and specific for the specific health care context. Specifically, this project relied heavily on the inclusion of hospital trauma care providers such as surgeons, emergency physicians, and nurses to review quality improvement data and suggest possible intervention strategies. This collaboration between the research team and local stakeholders permitted the intervention to be administered in a manner that maximized the likelihood of project acceptability and success.

While existing academic training programs such as ATLS can assist in care delivery standardization, the variability of LMIC trauma systems may decrease the applicability of high-income country (HIC) trainings in local LMIC communities [5,29]. For instance, teaching providers to obtain a chest x-ray or focused assessment with sonography in trauma (FAST) exam as part of the primary survey will only improve care outcomes if these providers have access to functional diagnostic imaging equipment. In a resource limited setting like Cameroon, diagnostic imaging is often not portable and located at a site far away from the trauma bay. Adherence to diagnostic imaging recommendations used in HICs would result in suboptimal trauma care as the patient would have to move away from the site of care to undergo diagnostic imaging. Likewise, while the number of patients who received cervical collar placement increased in the post-bundle cohort, about 85 % of patients meeting indications for cervical collar placement (i.e., unconscious patients, patients with numbness/weakness, or patients with more than one severe injury) still did not receive a cervical collar. This modest increase in cervical collar intervention reflects the broader reality in Cameroon that the limited availability of cervical collars can discourage providers from commonly applying them. Furthermore, LMIC providers may be limited by the compensation model of the local health system, as care may not be performed in a fee-for-service system until the patient pays out of pocket. The fee-for-service model in Cameroon is a significant barrier to emergency care provision that will not be resolved easily and organizing pre-packaged trauma kits in the ED works within existing compensation restraints to improve injury care. Though this incremental step in reducing a care barrier increased the amount of some interventions, there remains a great opportunity to further improve the quality of trauma care administered at this facility. The TQI bundle in this study does not represent a “one-size-fits-all” or replacement of existing resources in Cameroon. Instead, the method used to design this intervention leverages existing resources to adapt to the local context and optimize its efficacy.

This study contains several limitations. First, the observational cohort design using historical controls prevents the inference of causality between bundle implementation and outcomes. Rather, this intervention intended to generate baseline data regarding intervention efficacy to possible power a larger, multi-site bundle implementation trial in the future. Additionally, this study cannot evaluate the relative impact of the individual TQI bundle components on the associations with trauma practice measures. Finally, the relatively short time frame of this single-center study resulted in a small sample size. Thus, the study is likely underpowered to demonstrate differences in rare trauma outcomes, like emergency department death due to injury.

This intervention served as a first step in the development of a formal quality improvement system at this regional referral facility. Establishing a culture of quality improvement can allow this intervention to be sustainably continued over time. As quality improvement education increases and providers feel more empowered to provide quality care, patient outcomes are likely to improve and spur more improvement efforts. It was crucial to select a facility with administration and staff open to exploring quality improvement interventions. Since we have determined which interventions functioned well in this single-center setting, we aim to expand this quality improvement intervention to other hospitals affiliated with CTR data collection.

## Conclusion

The implementation of a context-appropriate TQI bundle was associated with significant improvements in previously identified trauma care deficits at a single regional hospital. Data-derived interventions targeting frontline capacity at the local level can bridge the gap between identifying care limitations and improvement in resource-limited settings.

## Supplementary Material

Supp Material 1

Supp Material 2

Supp Material 3

Supp Material 4

## Figures and Tables

**Table 1:** Demographic and injury severity of pre- and post-bundle implementation cohorts.

	Pre-intervention (*n* = 246) n (Percentage)	Post-intervention (*n* = 203) n (Percentage)	p-value
Age (median, IQR)	31 (22, 40)	34 (25, 48)	0.006*
Male sex	185 (75.2)	158 (77.8)	0.590
RTI mechanism	170 (69.1)	135 (66.5)	0.698
Missing	2 (0.8)	3 (1.5)	
HEAIS	3 (2,3)	3 (2,3)	0.637
Missing	2 (0.8)	1 (0.5)	
Complete set of vitals recorded	0	140 (69.0)	<0.001*
Time to vitals (mins, IQR)	5 (2, 8)	3 (0, 5)	<0.001*
Missing	25 (10.2)	10 (4.9)	
Vitals taken within 15 mins	181 (73.6)	183 (90.2)	<0.001*
Missing	25 (10.2)	10 (4.9)	
Initial measurement of HR and SBP	189 (76.8)	188 (92.6)	<0.001*
Repeat measurement of HR and SBP	17 (6.9)	106 (52.2)	<0.001*
Disposition			
Admit to ward/ICU	73 (29.7)	65 (32.0)	0.736
Admit directly to OR	16 (6.5)	4 (2.0)	0.030*
ED mortality	8 (3.3)	5 (2.5)	0.818
Missing	7 (2.9)	3 (1.5)	

Legend: *n* = number of patients, IQR = interquartile range, RTI = road traffic injury, HEAIS = highest estimated abbreviated injury scale, ICU = intensive care unit, OR = operating room, ED = emergency department, mins = minutes, HR = heart rate, SBP = systolic blood pressure.

* p-value less than 0.05.

**Table 2:** Recognition and treatment of primary survey abnormalities.

	Pre-intervention (*n* = 246) n (Percentage)	Post-intervention (*n* = 203) n (Percentage)	p-value
Patients with airway problem	1 (0.4)	16 (7.9)	<0.001*
Missing	2 (0.8)	0	
Patients with breathing problem	8 (3.3)	16 (7.9)	0.054
Missing	3 (1.2)	0	
Patients with circulation problem	185 (75.2)	146 (71.9)	0.408
Missing	2 (0.8)	0	
Patients with airway interventions	7 (2.9)	11 (5.4)	0.240
Missing	0	1.5 (3)	
Types of airway interventions			
Airway repositioning	6 (2.4)	8 (3.9)	0.522
Airway suctioning	0	7 (3.5)	0.008*
Non-invasive airway placement	0	2 (1.0)	0.408
Endotracheal intubation	1 (0.4)	0	1.000
Patients with breathing intervention	3 (1.2)	16 (7.9)	<0.001*
Missing	4 (1.6)	4 (2.0)	
Types of breathing interventions			
Oxygen via non-BVM	3 (1.2)	15 (7.4)	0.002*
Oxygen via BVM	1 (0.4)	2 (1.0)	0.856
Needle thoracostomy	0	2 (1.0)	0.408
Patients with circulation intervention	229 (93.1)	182 (89.7)	0.396
Missing	17 (6.9)	19 (9.4)	
Types of circulation interventions			
Tourniquet placed	10 (4.1)	4 (2.0)	0.318
Blood transfusion	17 (6.9)	13 (6.4)	0.986
IV crystalloid or colloid fluid	226 (91.9)	181 (89.2)	0.412
Patients with c-collar placement in ED	0	6.9 (14)	<0.001*
Missing	2 (0.8)	0	
Patients with indications for c-collar	85 (34.6)	91 (44.8)	0.034*

Please note that the number of interventions may be greater than the number of patients because multiple interventions may have been performed on the same patient.

Legend: *n* = number of patients, BVM = bag-valve-mask, C-collar = cervical spine collar, mins = minutes.

* p-value less than 0.05.

## References

[1] Charles AG, Mock C. Advancing global surgery: moving beyond identifying problems to finding solutions. World J Surg 2017;41:2979–80. 10.1007/s00268-017-4316-9. 29067518

[2] Gosselin RA, Spiegel DA, Coughlin R, Zirkle LG. Injuries: the neglected burden in developing countries. Bull World Health Organ 2009;87:246. 10.2471/BLT.08.052290. 19551225 PMC2672580

[3] World Health Organization. Injuries and violence: the facts 2014. Geneva: World Health Organization; 2014.

[4] Mock C, Joshipura M, Arreola-Risa C, Quansah R. An estimate of the number of lives that could be saved through improvements in trauma care globally. World J Surg 2012;36:959–63. 10.1007/s00268-012-1459-6. 22419411

[5] Jayaraman S, Sethi D. Advanced trauma life support training for hospital staff. The Cochrane Collaboration, editor. Cochrane database of systematic reviews. Chichester, UK: John Wiley & Sons, Ltd; 2009, CD004173. 10.1002/14651858.CD004173.pub3.. pub3. 19370594

[6] South SD, Boeck MA, Foianini JE, Swaroop M. Advanced trauma life support preparatory courses in low- and middle-income countries. J Am Coll Surg 2017; 225:S98. 10.1016/j.jamcollsurg.2017.07.214.

[7] Kornfeld JE, Katz MG, Cardinal JR, Bat-Erdene B, Jargalsaikhan G, Nunez J. Cost analysis of the Mongolian ATLS© program: a framework for low- and middle-income countries. World J Surg 2019;43:353–9. 10.1007/s00268-018-4795-3. 30353403

[8] The World Bank. World bank country and lending groups. The World Bank; 2023. https://datahelpdesk.worldbank.org/knowledgebase/articles/906519-world-bank-country-and-lending-groups.

[9] Christie SA, Dickson D, Mbeboh SN, Embolo FN, Chendjou W, Wepngong E, Association of health care use and economic outcomes after injury in Cameroon. JAMA Netw Open 2020;3. 10.1001/JAMANETWORKOPEN.2020.5171. e205171–e205171. 32427321 PMC7237963

[10] Juillard CJ, Stevens KA, Ekeke M, Georges M, Etoundi A, Marquise M, Analysis of prospective trauma registry data in francophone Africa: a pilot study from Cameroon. World J Surg 2014;38:2534–42. 10.1007/s00268-014-2604-1. 24791906

[11] Nwanna-Nzewunwa OC, Christie SA, Carvalho M, Motwani G, Dissak Delon FN, Ngamby MK, Analysis of a national trauma registry in Cameroon: implications for prehospital care strengthening. Panam J Trauma Crit Care Emerg Surg 2018;7:133–42. 10.5005/JP-JOURNALS-10030-1216.

[12] Cameroon Ministry of Public Health, World Health Organization. Tracking 100 core health indicators in Cameroon in 2019 & SDG focus. 2019. Yaoundé, Cameroon.

[13] Christie SA, Zheng D, Dissak-Delon F, Kinge T, Njock R, Nkusu D, How trauma patients die in low resource settings: identifying early targets for trauma quality improvement. J Trauma Acute Care Surg 2022. 10.1097/TA.0000000000003768. PMC9877108 36163642

[14] Ding K, Sur PJ, Mbianyor MA, Carvalho M, Oke R, Dissak-Delon FN, Mobile telephone follow-up assessment of postdischarge death and disability due to trauma in Cameroon: a prospective cohort study. BMJ Open 2022;12:56433. 10.1136/bmjopen-2021-056433. PMC8984008 35383070

[15] Koster J, Hassall OW. Attitudes towards blood donation and transfusion in Bamenda, Republic of Cameroon. Transfusion Med 2011;21:301–7. 10.1111/j.1365-3148.2011.01079.x. 21762226

[16] Tagny CT. Status of blood transfusion safety in Cameroon. Transfusion Apheresis Sci 2023:103800. 10.1016/j.transci.2023.103800. 37661489

[17] Kindzeka ME Cameroon begs civilians to donate blood on world blood donor day. Voice of America News 2021. https://www.voanews.com/a/africa_cameroon-begs-civilians-donate-blood-world-blood-donor-day/6207013.html.

[18] Kindzeka ME Cameroon officials campaign against taboos to encourage people to donate blood. Voice of America News 2023. https://www.voanews.com/a/cameroon-officials-campaign-against-taboos-to-encourage-people-to-donate-blood/7135283.html.

[19] World Health Organization. WHO trauma care checklist 2016. https://www.who.int/publications-detail-redirect/trauma-care-checklist.

[20] Primary trauma care foundation 2015. https://www.primarytraumacare.org.

[21] Kampala advanced trauma care course 2024. https://www.kampalatrauma.com/.

[22] World Health Organization, International Committee of the Red Cross. WHO-ICRC basic emergency care: approach to the acutely ill and injured 2018. https://www.who.int/publications-detail-redirect/9789241513081 (accessed January 10, 2024).

[23] Michie S, Van Stralen MM, West R. The behaviour change wheel: a new method for characterising and designing behaviour change interventions. Implement Sci 2011; 6:42. 10.1186/1748-5908-6-42. 21513547 PMC3096582

[24] Ogrinc G, Davies L, Goodman D, Batalden P, Davidoff F, Stevens D. SQUIRE 2.0 (Standards for QUality Improvement Reporting Excellence): revised publication guidelines from a detailed consensus process. BMJ Qual Saf 2016;25:986–92. 10.1136/bmjqs-2015-004411. PMC5256233 26369893

[25] Yates F Tests of significance for 2 × 2 contingency tables. R Stat Soc J Ser A: General 1984;147:426–49. 10.2307/2981577.

[26] StataCorp. Stata statistical software: release 16. 2019.

[27] Yost MT, Carvalho MM, Mbuh L, Dissak-Delon FN, Oke R, Guidam D, Back to the basics: clinical assessment yields robust mortality prediction and increased feasibility in low resource settings. PLoS Glob Public Health 2023;3:e0001761. 10.1371/journal.pgph.0001761. 36989211 PMC10057736

[28] Petroze RT, Byiringiro JC, Ntakiyiruta G, Briggs SM, Deckelbaum DL, Razek T, Can focused trauma education initiatives reduce mortality or improve resource utilization in a low-resource setting? World J Surg 2015;39:926–33. 10.1007/s00268-014-2899-y. 25479817 PMC4700401

[29] Brown HA, Tidwell C, Prest P. Trauma training in low- and middle-income countries: a scoping review of ATLS alternatives. Afr J Emerg Med 2022;12:53–60. 10.1016/j.afjem.2021.11.004. 35070655 PMC8761604

