# Supplementary material for: From targets to solutions: Implementing a trauma quality improvement bundle in Cameroon
Source: Injury. Author manuscript; Available in PMC 2025 Apr 20. (PMC12009632; doi:10.1016/j.injury.2024.111625)
Supplement: Supp Material 1 [file NIHMS2073023-supplement-Supp_Material_1.pdf]

Liste de contrôle des soins de traumatisme [redacted] *[1.0]* date d'arrivée du patient: / /

- [ ] **Triage** et **transport** appropriés des patients

- [ ] Évaluation des voies **Aériennes**
- [ ] si nécessaire Intervention sur les voies **Aériennes (de base)** effectuée: ____________________
- [ ] si nécessaire Intervention sur les voies **Aériennes (avancée/définitive)** demandée: ________
- [ ] si nécessaire Immobilisation de la **colonne vertébrale cervicale**
- [ ] Vérification des **signes vitaux** (complets)
- [ ] Evaluation de la **Respiration** (générale, auscultation)
- [ ] si nécessaire Intervention **Respiratoire (oxygène)** effectuée
- [ ] si nécessaire Intervention **Respiratoire (avancée)** effectuée/demandée: ____________________
- [ ] Évaluation de la **Circulation** sanguine
- [ ] si nécessaire Intervention **circulatoire (fluides IV)** réalisée
- [ ] si nécessaire Intervention **circulatoire (contrôle de l'hémorragie)** effectuée: _______________
- [ ] Évaluation **Neurologique** (GCS, des pupilles) effectuée
- [ ] Évaluation de l'état **neurovasculaire** des extrémités
- [ ] Le patient est **Exposé** à des blessures cachées
- [ ] **Évaluation et gestion de toutes les menaces immédiates pour la vie**

- [ ] **Antécédents** pertinents obtenus

- [ ] Evaluation secondaire : **Tête, visage**
- [ ] Evaluation secondaire : **Cou**
- [ ] Evaluation secondaire : **Poitrine**
- [ ] Evaluation secondaire : **Abdomen**
- [ ] Evaluation secondaire : **Pelvis, organes génitaux**
- [ ] Evaluation secondaire : **Extrémités**
- [ ] Evaluation secondaire : **Dos, colonne vertébrale**

- [ ] si nécessaire **Imagerie** demandée: ____
- [ ] si nécessaire **Anesthésie** consultée
- [ ] si nécessaire **Chirurgie** consultée: ____________________
- [ ] si nécessaire **Médicaments** (tétanos, antibiotiques, analgésie): ____________________
- [ ] si nécessaire **Tests de laboratoire** demandés: ________________________________
- [ ] si nécessaire **Transfert demandé**
- [ ] si nécessaire **Examens en série** (neurologiques, abdominaux, vasculaires): ________________
- [ ] **Kit d'urgence** utilisé

Trauma Care Checklist [1.0]   Patient arrival date: / /

☐ Proper patient **triage** and **transport**

☐ **Airway** assessment
☐ (If necessary) **Airway** intervention (**basic**) carried out: ______________________________
☐ (If necessary) **Airway** intervention (**advanced/definitive**) requested: __________________
…..
☐ (If necessary) Immobilization of the **cervical spine**
☐ Verification of **vital signs** (complete)
…..
☐ **Breathing** Assessment (<u>general, auscultation</u>)
☐ (If necessary) **Respiratory** Intervention (**oxygen**) carried out
☐ (If necessary) **Respiratory** intervention (**advanced**) carried out/requested: _____________
…..
☐ Assessment of blood **Circulation**
☐ (If necessary) **Circulatory** intervention (**IV fluids**) performed
☐ (If necessary) **Circulatory** intervention (**hemorrhage control**) performed: ______________
…..
☐ **Neurological** Assessment (<u>GCS, pupils</u>) carried out
☐ Assessment of **neurovascular** status of extremities
…..
☐ The patient is **exposed** to reveal hidden injuries
☐ **Assessment and management of all immediate threats to life**

☐ Relevant **history** obtained

☐ Secondary evaluation: **Head, face**
☐ Secondary evaluation: **Neck**
☐ Secondary evaluation: **Chest**
☐ Secondary evaluation: **Abdomen**
☐ Secondary evaluation: **Pelvis, genital organs**
☐ Secondary evaluation: **Extremities**
☐ Secondary evaluation: **Back, spine**

☐ (If necessary) **Imagery** requested:_______________________
☐ (If necessary) **Anesthesia** consulted ☐ (If necessary) **Surgery** consulted: _____________

☐ (If necessary) **Medications** (tetanus, antibiotics, analgesics): ___________________________
☐ (If necessary) **Laboratory tests** requested: ____
☐ (If necessary) **Transfer requested**
☐ (If necessary) **Serial examinations** (neurological, abdominal, vascular): __________________
☐ **Emergency kit** used
