# Supplementary material for: From targets to solutions: Implementing a trauma quality improvement bundle in Cameroon
Source: Injury. Author manuscript; Available in PMC 2025 Apr 20. (PMC12009632; doi:10.1016/j.injury.2024.111625)
Supplement: Supp Material 2 [file NIHMS2073023-supplement-Supp_Material_2.docx]

Beginning May 2, 2022,

The hospital will guarantee the presence of a supply kit for the initial care of trauma patients in the Emergency Department.

This kit will contain the following supplies, which are necessary for the acute care of each trauma patient without delay:

- 5 pairs of non-sterile examination gloves / *Gants de soins non sterilizes* no 7,5/8 – each costs 200 Central African Francs (CFA)
- 2 IV cannula (Catheter N18 – each costs 250 CFA), 2 seringue aiguille 10 mL (each costs 75), 2 500cc saline (each costs 750 CFA), 2 perfuseur/drip set (each costs 250 CFA)
- Gauze compresses / Compresses steriles 10x10cm, 1 packet / 10 feuilles – each costs 700 CFA
- Bandages / Sparadrap perfore (costs 350 CFA)
- Crepe bandage / bande velpeau (costs 500 CFA)

| For each patient: | Qty | Unit cost | Total cost for 1 patient |
| --- | --- | --- | --- |
| Non-sterile examination gloves (*Gants de soins non sterilizes*) | 5 | 200 CFA | 1000 CFA |
| Catheter N18 | 2 | 250 CFA | 500 CFA |
| Syringe needle (*Seringue aiguille*) | 2 | 75 CFA | 150 CFA |
| 500cc saline | 2 | 750 CFA | 1500 CFA |
| Perfuseur/drip set | 2 | 250 CFA | 500 CFA |
| Sterile packet gauze compresses (*Compresses steriles*) | 1 | 700 CFA | 700 CFA |
| Bandages (*Sparadrap perfore*) | 1 | 350 CFA | 350 CFA |
| Crepe bandage (*Bande velpeau*) | 1 | 500 CFA | 500 CFA |
| 5200 CFA total | | | |

Total for 10 patients at one time

**Storage cabinet must contain:**

- 50 Non-sterile examination gloves (*gants de soins non sterilizes*)
- 20 Catheter N18
- 20 Syringe needle (*Seringe aiguille*)
- 20 500cc saline
- 20 Perfuseur/drip set
- 10 Packets sterile Gauze compresses (*compresses sterile*)
- 10 Bandages (*Sparadrap perfore*)
- 10 Crepe bandage (*Bande velpeau*)

The kit is to be stored in the cabinet in the Treatment Room (*Salle de Soins*). The nurse major will be responsible for replenishing it from the pharmacy each day.

The kit should be used only for patients presenting with traumatic injury necessitating treatment.

Each time a patient with a traumatic injury arrives, the staff should use the supplies in the kit to provide initial care for the patient according to the basic trauma protocol.

The cost of the kit for each patient (5200 CFA) should then be added to the patient’s bill, which will be discussed with the hospital cashier after initial care has been provided to the patient.
