# Supplementary material for: From targets to solutions: Implementing a trauma quality improvement bundle in Cameroon
Source: Injury. Author manuscript; Available in PMC 2025 Apr 20. (PMC12009632; doi:10.1016/j.injury.2024.111625)
Supplement: Supp Material 3 [file NIHMS2073023-supplement-Supp_Material_3.docx]

Trauma Quality Improvement Interventions Alongside Relevant COM-B Components and BCW Intervention Functions

| **Bundle component** | **Target behavior** | **Barriers to behavior** | **Function of component** | **COM-B component(s)** | **Intervention function(s)** |
| --- | --- | --- | --- | --- | --- |
| Trauma protocol | Appropriately recognize and intervene on specific problems in injured patients | Lack of standardized guidelines for trauma in local setting | Development of contextually appropriate set of guidelines for trauma care | Psychological capability | Education, Restriction |
| Trauma checklist | Systematically assess injured patients from head to toe, prioritizing threats to life | Variations in provider knowledge and capabilities, trauma patient volume | Development of physical decision-making tool to remind providers of key steps in care | Physical capability, Psychological capability | Environmental restructuring, Enablement |
| Trauma training | Draw upon awareness and knowledge of trauma care principles | Lack of established training for trauma in local setting | Provision of comprehensive training program for trauma care | Psychological capability | Education, Training |
| Emergency kit | Obtain and utilize emergency care supplies in time-efficient manner | Delays associated with procurement of supplies from hospital pharmacy | Development of basic emergency care kit to be safely stored in emergency department | Physical opportunity | Environmental restructuring, Enablement |
| Quality audit meeting | Continuously analyze data to improve clinical performance and enhance quality improvement culture | Lack of access to performance data, lack of opportunities to review adherence to guidelines | Institution of monthly meeting to review newly created quality improvement reports | Social opportunity, Reflective motivation | Education, Persuasion, Modelling |
