# Supplementary material for: From targets to solutions: Implementing a trauma quality improvement bundle in Cameroon
Source: Injury. Author manuscript; Available in PMC 2025 Apr 20. (PMC12009632; doi:10.1016/j.injury.2024.111625)
Supplement: Supp Material 4 [file NIHMS2073023-supplement-Supp_Material_4.docx]

| **Weekly period** | **Number of trauma checklists completed** | **Number of emergency kits used** |
| --- | --- | --- |
| June 3 to June 8 | 13 | 9 |
| June 9 to June 15 | 16 | 11 |
| June 16 to June 20 | 13 | 7 |
| June 21 to June 27 | 15 | 11 |
| June 28 to July 4 | 22 | 8 |
| July 5 to July 11 | 18 | 14 |
| July 12 to July 18 | 12 | 6 |
| July 19 to July 25 | 15 | 5 |
| July 26 to August 1 | 12 | 7 |
| August 2 to August 8 | 30 | 13 |
| August 9 to August 15 | 15 | 9 |
| August 16 to August 22 | 23 | 5 |
| August 23 to August 29 | 5 | 10 |
| August 30 to September 5 | 7 | 0 |
| September 6 to September 12 | 6 | 1 |
| September 13 to September 19 | 20 | 4 |
| September 20 to September 26 | 16 | 0 |
| September 27 to October 3 | 21 | 0 |
| October 4 to October 10 | 11 | 1 |
| October 11 to October 17 | 27 | 9 |
| October 18 to October 24 | 13 | 7 |
| October 25 to October 31 | 10 | 2 |
| November 1 to November 7 | 18 | 2 |
| November 8 to November 14 | 18 | 1 |
| November 15 to November 21 | 17 | 0 |
| November 22 to November 28 | 7 | 0 |
